# Alterations of T cell activation signalling and cytokine production by postmenopausal estrogen levels

**DOI:** 10.1186/1742-4933-6-1

**Published:** 2009-03-05

**Authors:** Lowell T Ku, Cicek Gercel-Taylor, Steven T Nakajima, Douglas D Taylor

**Affiliations:** 1 Division of Reproductive Endocrinology and Infertility, Department of Obstetrics, Gynecology and Women's Health, University of Louisville School of Medicine, Louisville, Kentucky, USA

## Abstract

**Background:**

Immunosenescence is an age-associated disorder occurring primarily in T cell compartments, including altered subset composition, functions, and activation. In women, evidence implicates diminished estrogen in the postmenopausal period as a contributing factor to diminished T cell responsiveness. Since hypoestrogenism is present in postmenopausal women, our objective focused on whether T cell activation, defined as signalling molecule expressions and activation, and function, identified as IL-2 production, were affected by low estrogen.

**Methods:**

Using Jurkat 6.1 T cells, consequences of 4 pg/ml (corresponding to postmenopausal levels) or 40 pg/ml (premenopausal levels) of estradiol (E_2_) were analyzed on signalling proteins, CD3-zeta, JAK2, and JAK3, determined by Western immunoblotting. These consequences were correlated with corresponding gene expressions, quantified by real time-polymerase chain reaction. Tyrosine phosphorylation of CD3-zeta was defined by immunoprecipitation and western immunoblotting following activation by T cell receptor (TcR) cross-linking. CD3-zeta expression and modulation was also confirmed in T cells from pre- and postmenopausal women. To assess functional consequences, IL-2 production, induced by PMA and ionomycin, was determined using enzyme-linked immunosorbent spot assay (ELISpot).

**Results:**

At 40 pg/ml E_2_, the level of signalling protein CD3-zeta was elevated 1.57-fold, compared with cells exposed to 4 pg/ml E_2_. The CD3-zeta proteins also exhibited altered levels of activation-induced phosphorylation in the presence of 40 pg/ml E_2 _versus 4 pg/ml: 23 kD phosphorylated form increased 2.64-fold and the 21 kD form was elevated 2.95-fold. Examination of kinases associated with activation signalling also demonstrated that, in the presence of 40 pg/ml E_2_, JAK2 protein expression was increased 1.64-fold (p < 0.001) and JAK3 enhanced 1.79-fold (p < 0.001) compared to 4 pg/ml. mRNA levels for CD3-zeta, JAK2, and JAK3 were significantly increased following exposure to 40 pg/ml E_2 _(2.39, 2.01, and 2.21 fold, respectively) versus 4 pg/ml. These findings were confirmed in vivo, since T cells from postmenopausal women exhibited 7.2-fold diminished CD3-zeta expression, compared to pre-menopausal controls and this expression was elevated 3.8-fold by addition of 40 pg/ml E_2_. Functionally, Jurkat cells exposed to 40 pg/ml E_2 _and activated exhibited significantly elevated numbers of IL-2 producing colonies compared to 4 pg/ml (75.3 ± 2.2 versus 55.7 ± 2.1 colonies, p < 0.0001).

**Conclusion:**

Jurkat T cells exposed to 4 pg/ml E_2 _expressed significantly diminished activation signalling proteins, correlating with reduced IL-2 production. Lower signalling protein levels appear to result from decreased CD3-zeta, JAK2, and JAK3 gene expressions. These findings may provide a molecular basis for immunosenescence associated with the postmenopausal state.

## Background

Immunosenescence, the gradual deterioration of immune responsiveness is one of the age-associated phenomena observed in humans [1,2]. While age-dependent defects are seen in many cell types leading to immunosenescence, defects in T cell function are the most dramatic and consistently observed and are generally responsible for aberrations in protective immunity at both the cellular and humoral levels [3,4]. Assessment of T cell responses from elderly humans have indicated a dysregulation of intracellular signal transduction capacities, reduced diversity of the antigen recognition repertoire of T cell receptors, impaired proliferation in response to antigenic stimulation (antigens, mitogenic lectins, or antibodies directed against CD3 components), and changes in cytokine profiles [5-11]. These age-associated immunological changes make an individual more susceptible to infection, cancer, age-associated autoimmune diseases, and, indirectly, to atherosclerosis and Alzheimer's disease [12].

Two major changes in human T cell functions associated with aging are diminished proliferation and decreased secretion of interleukin-2 (IL-2) after activation via the T cell receptor (TcR)/CD3 complex [5,13,14], although the molecular mechanisms for these changes are not well understood. Alterations in intracellular signalling transduction have been postulated to mediate functional defects exhibited by senescent lymphocytes [15]. Several studies have suggested that aberrancies in early TcR/CD3 mediated signalling events may contribute to T cell function decline during aging [6,9,14,16]. CD3-zeta chains are associated with the T cell receptor complex and generate an activation signal in T lymphocytes in response to cross-linking of antigen-binding sites by antigen or mitogens [8,17]. Janus kinases (JAK) are a family of non-receptor tyrosine kinases that transduce cytokine-mediated signals via the JAK-STAT pathway in response to the IL-2 cytokine activating the IL-2 receptor [17]. During T cell activation, cytokines are produced that play a pivotal role in cellular growth, differentiation and apoptosis. In order for signal transduction of cytokine receptors to reach the nucleus, two different types of molecules play important roles: Janus kinases (JAK-1,-2,-3 and Tyk-2) and signal transducers and activators of transcription (STAT) proteins (STAT-1,2,3,4,5,6). Phosphorylation of JAKs takes place following the binding of cytokines and leads to activation of STAT proteins.

Although immunosenescence affects both men and women, it does not affect them equally. Men (all ages) and postmenopausal women exhibit diminished T cell immunity compared to premenopausal women [18]. The decrease in androgens in men with aging may contribute to their immunosenescence; however, the loss of T cell function in men with aging is significantly less dramatic than that observed in women [19-21]. Estrogen is not a single component, but consists of multiple forms; the primary circulating forms are estrone (E_1_), estradiol (E_2_) and estriol (E_3_). Estradiol binds both estrogen receptor-(ER)α and ERβ with high and equal affinities, while estrone preferentially binds ERα at a 5-fold higher affinity than ERβ [22]. Recent studies have demonstrated that, while ERα and ERβ exhibit distinct functions within immune cells, both pathways are involved in mediating estrogen effects [23]. In premenopausal women, the principal circulating estrogen is ovary-derived estradiol, while in postmenopausal women and men, estrone is the most abundant circulating estrogen. In men, testosterone, which exhibits a small age-related decrease, is the primary substrate for estrogen production by peripheral aromatization of androgens precursors; however, most studies failed to observe any significant influence of age on total E_2 _levels in men [21]. Since our study focuses on the menopause-linked association of the loss of estradiol and T cell activation, the role of estrogens as an immunoregulator in men is not addressed.

In women, many studies have demonstrated that the presence of estrogens is necessary for a robust immune response to pathogens [24-27]; however, the more active immune system in females can also lead to a predominance of diverse autoimmune diseases. In estrogen-deficient states, such as menopause or surgical castration; the immune system produces a blunted response. The strength of the immune response in the presence and absence of estrogen has been demonstrated in the murine model [28,29]. Young female proestrus mice exhibited enhanced immune responses compared to their male counterparts, with estradiol levels appearing to be responsible for the enhanced immune response. Aged female rodents exhibited a reduced T lymphocyte response compared to young female rodents when exposed to trauma-hemorrhage [28]. Additionally, surgically castrated female rats produced a diminished mitogen-induced T cell proliferation response as well as decreased lymphocyte chemotaxis and IL-2 production compared to non-castrated female rats [29]. These findings from murine models are consistent with data collected from humans. Postmenopausal women have also been shown to have diminished immune responses. Estrogen-deficient women have been shown to have poor anti-viral responses [25], reduced proliferation of lymphocytes [7], exhibit reductions in B cells and T helper cells [24], as well as T helper cell-derived cytokines [27].

Since decreased levels of estrogens are associated with the postmenopausal state and correlate with immunosenescence, our hypothesis for an underlying mechanism of blunted T cell responses exhibited in postmenopausal women is that the diminished production of Th1 cytokines, such as IL-2, is due to decreased activation signalling components, resulting from estrogen deficiency. This hypothesis is supported by the observation that estrogen replacement treatment has been demonstrated to restore T cell functions [26]. The objectives of this study were to determine whether Th1 function, specifically IL-2 production, by an immortalized line of T lymphocytes was diminished in a low estrogen environment and to determine whether this decreased IL-2 production was attributable to decreased gene transcription or decreased mRNA translation of modulators of signaling components, CD3-zeta, JAK 2, and JAK 3.

## Results

### Modulation of TCR activation signalling

**Figure 1:**
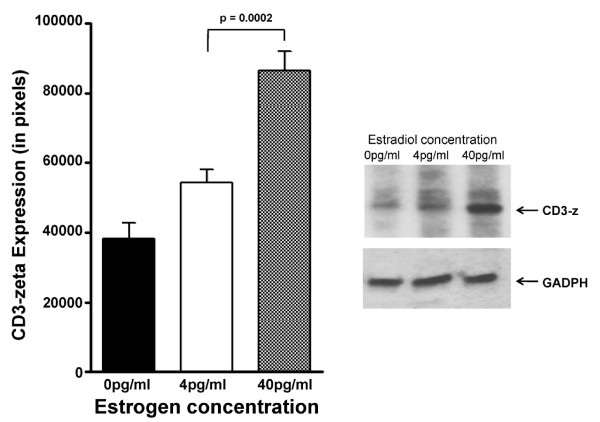
**Modulation of Jurkat cell CD3-zeta protein expression by physiologic estradiol concentrations**. Expression of CD3-zeta protein expression by Jurkat cells incubated in the presence of either 4 pg/ml or 40 pg/ml estradiol for 48 hours defined by Western immunoblotting. The bar graph presents the quantitation of triplicate digitized gel images expressed as mean ± standard deviation. The insert shows a representative Western blot gel image.

**Figure 2:**
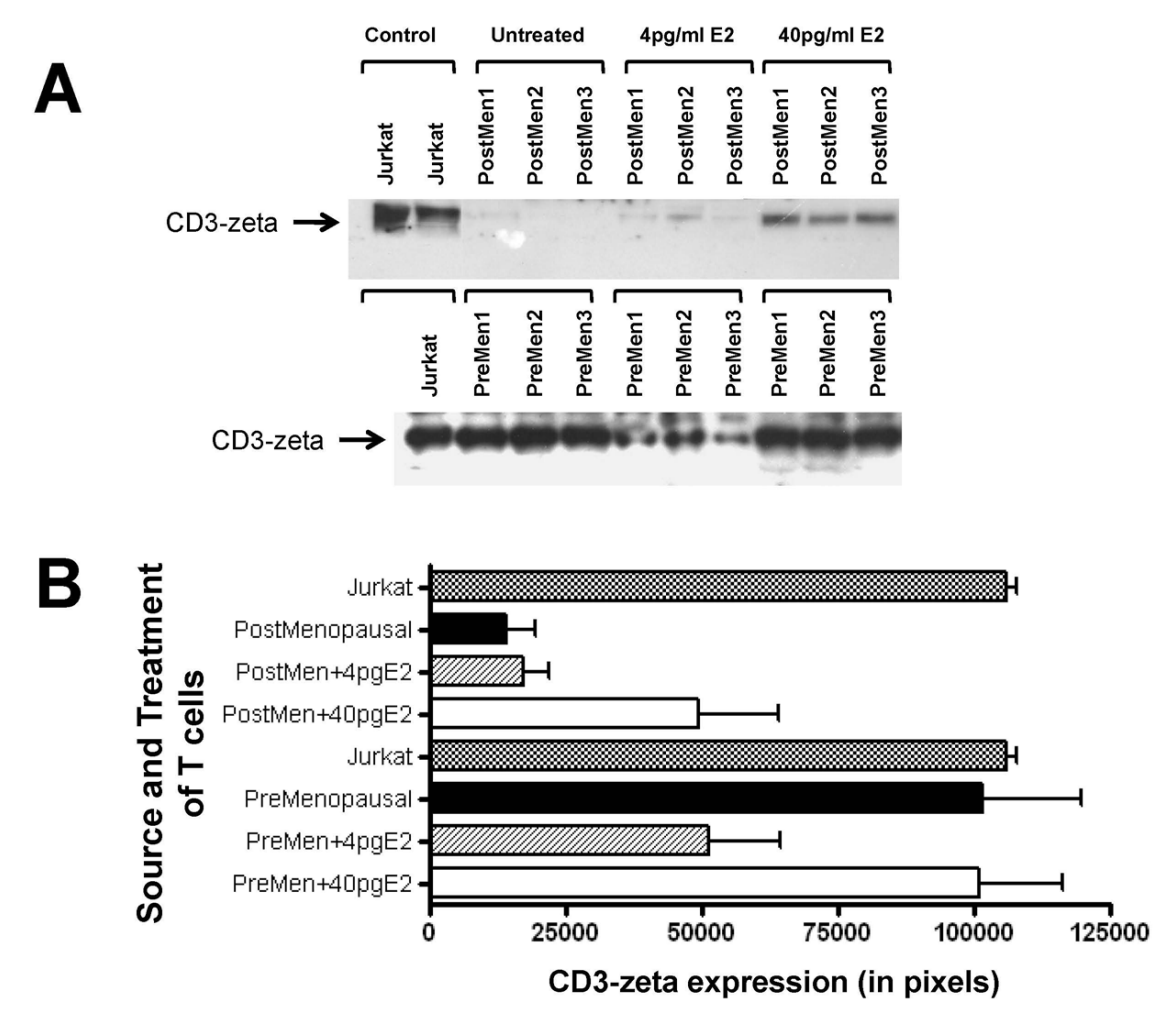
**Modulation of normal T cell CD3-zeta protein expression by physiologic estradiol concentrations**. Expression of CD3-zeta protein expression by T cells isolated from either normal premenopausal or postmenopausal female volunteers, assayed immediately after isolation and following an incubation in the presence of either 4 pg/ml or 40 pg/ml estradiol for 48 hours defined by Western immunoblotting. The bar graph presents the quantitation of triplicate digitized gel images expressed as mean ± standard deviation. The insert shows a representative Western blot gel image.

To define the consequences of estradiol concentrations on cellular levels of activation signalling proteins, Jurkat cells were incubated in 4 pg/ml or 40 pg/ml of E_2 _and the relative levels of cellular CD3-zeta protein were determined by Western immunoblotting. In the presence of 4 pg/ml E_2_, CD3-zeta protein levels were 1.57-fold lower, compared to CD3-zeta protein expressed in cells incubated in 40 pg/ml (Figure 1). This difference was statistically significant (p < 0.001). Since Jurkat cells may not represent the optimal model to investigate the consequence of menopausal estrogen decline, these results indicating diminished CD3-zeta levels in the postmenopausal environment were confirmed using T cells isolated from pre and postmenopausal women (Figure 2). When defined immediately after isolation, CD3-zeta expression was 7.2-fold lower in the postmenopausal group compared to premenopausal women. When T cells from postmenopausal women were incubated with 4.0 pg/ml E_2_, no significant change was observed; however, when incubated with 40.0 pg/ml E_2 _for 48 hours, a 3.5-fold increase in CD3-zeta expression was observed. In contrast, when T cells from premenopausal women were incubated with 4.0 pg/ml E_2 _for 48 hours, a 2.0-fold decrease in CD3-zeta expression was demonstrated.

**Figure 3:**
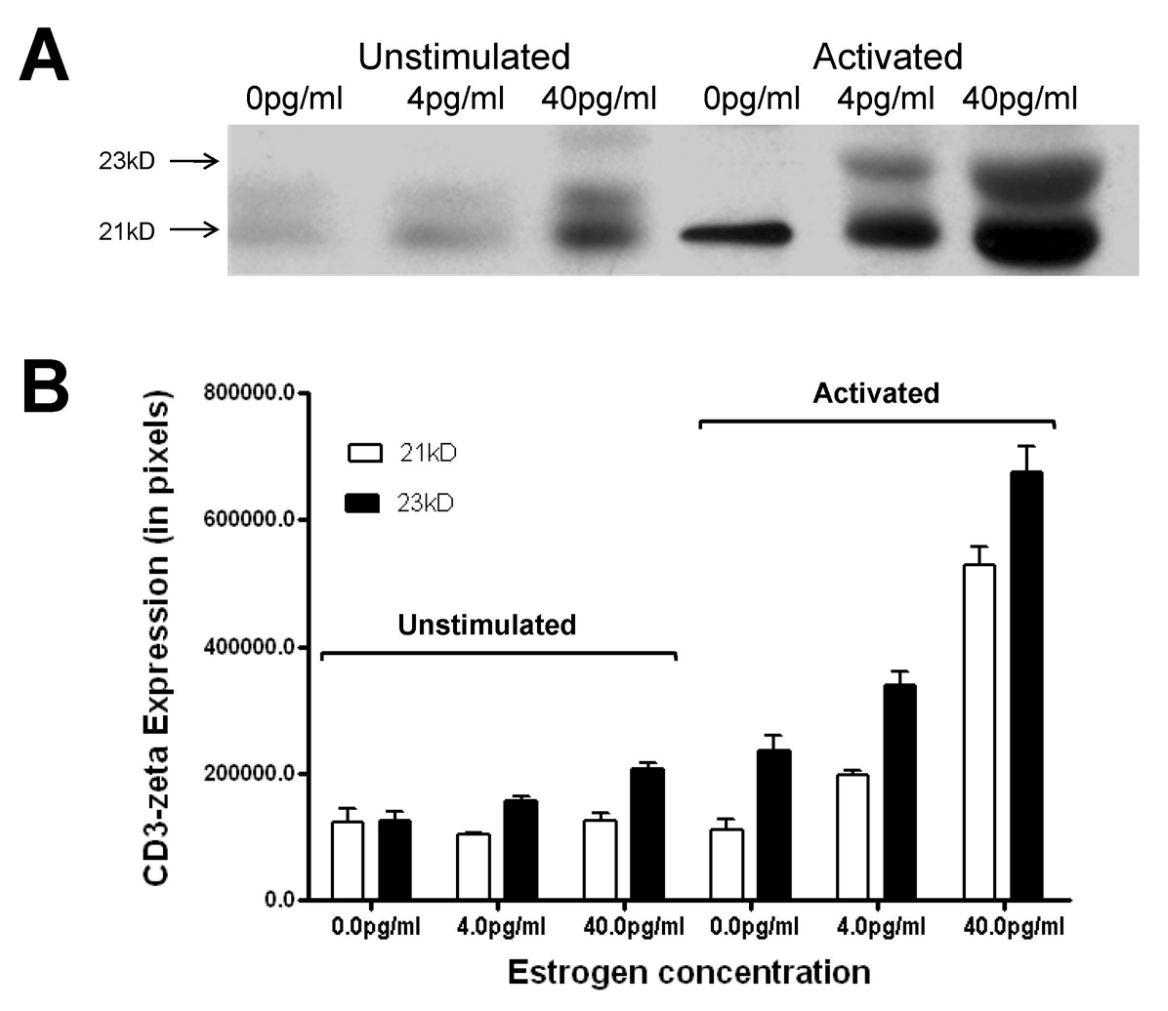
**Activation-induced phosphorylation of CD3-zeta at physiologic estradiol concentrations**. Representative western immunoblotting results demonstrating the major phosphorylated CD3-zeta bands. Jurkat cells were incubated for 48 hours in 0, 4 or 40 pg/ml estradiol and then activated by cross-linking the TcR. Cellular CD3-zeta was immunoprecipitated and the phosphorylated bands were identified using anti-pCD3-zeta as the primary antibody.

The earliest intracellular event to occur in response to TcR binding of antigen is phosphoryl-tyrosine kinase-mediated phosphorylation of the immunoreceptor tyrosine activation motifs (ITAMs) of the CD3-zeta chains. The phosphorylation of CD3-zeta protein was examined following activation by cross-linking the TcR (Figure 3). Two prominent phosphorylated bands were observed following activation: 21 kD and 23 kD. In the presence of 40 pg/ml E_2_, these two bands exhibited enhanced levels of phosphorylation, compared to their counterparts at 4 pg/ml. After adjusting to equal CD3-zeta protein, the 21 kD band was observed to exhibit a 2.95-fold greater level of phosphorylation and the 23 kD was shown to be 2.64-fold greater.

### Modulation of activation-associated kinase expression

**Figure 4:**
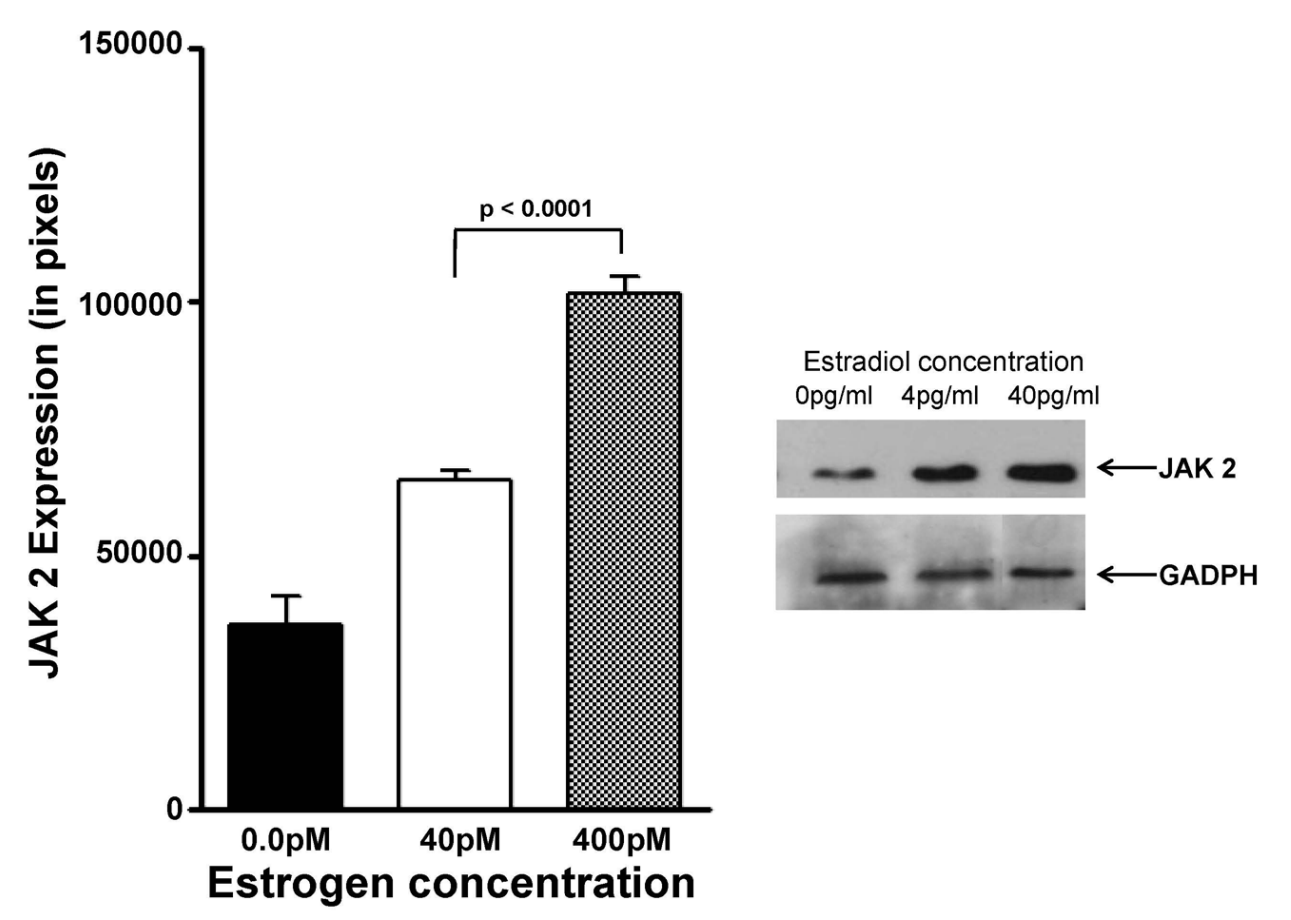
**Modulation of JAK 2 protein expression by physiologic estradiol concentrations**. Modulation of JAK 2 protein expression in Jurkat cells incubated in the presence of either 4 pg/ml or 40 pg/ml estradiol for 48 hours defined by Western immunoblotting. The bar graph shows the quantitation of triplicate digitized gel images expressed as mean ± standard deviation. The insert shows a representative Western blot gel image.

**Figure 5:**
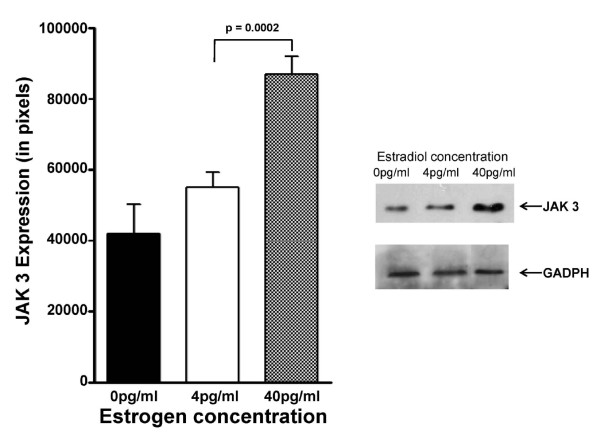
**Modulation of JAK 3 protein expression by physiologic estradiol concentrations**. Modulation of JAK 3 protein expression in Jurkat cells incubated in the presence of either 4 pg/ml or 40 pg/ml estradiol for 48 hours defined by Western immunoblotting. The bar graph presents the quantitation of triplicate digitized gel images expressed as mean ± standard deviation. The insert shows a representative Western blot gel image.

The effects of E_2 _levels of the expression of other components associated with non-TcR mediated signalling, specifically JAK 2 and JAK 3, were then examined. JAK2 is a non-receptor type of protein tyrosine kinase associated with the intracellular domains of cytokine receptors, including IL-3, GM-CSF and erythropoietin. Jurkat cells, exposed to 4 pg/ml estradiol, expressed 1.64-fold less JAK 2 when compared to those in 40 pg/ml estradiol (Figure 4, which was statistically significant (p < 0.001). JAK 3 is a non-receptor type tyrosine kinase, involved in the signaling of interleukins containing the γ c chain, such as IL-2, IL-4, IL-7, IL-9, IL-15, and IL-21. JAK 3 protein expression (Figure 5) was 1.79-fold lower in Jurkat cells incubated with 4 pg/ml estradiol versus Jurkat cells incubated with 40 pg/ml (p < 0.0001). For each of these proteins, their levels in 4 pg/ml were significantly greater than that observed in 0 pg/ml estradiol: for JAK 2, p < 0.001; and JAK 3, p < 0.05.

### Consequences of estrogen on gene expression of signalling components

**Figure 6:**
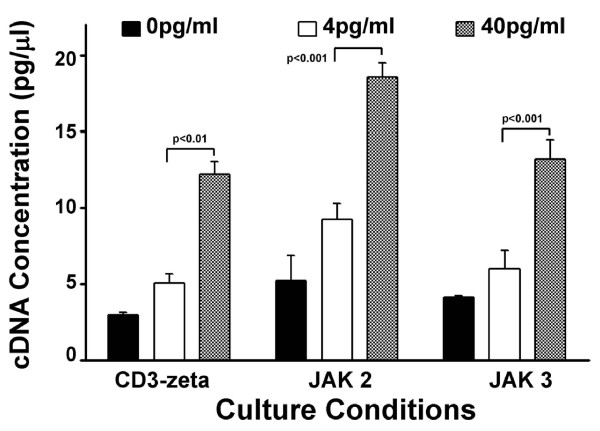
**Suppression of CD3-zeta, JAK 2 and JAK 3 mRNA levels by physiologic estradiol concentrations**. Suppression of mRNA levels for CD3-zeta, JAK 2, and JAK 3 by exposure to 4 pg/ml versus 40 pg/ml estradiol quantified by real-time PCR. Results are presented as a mean ± standard deviation of cDNA concentration, resulting from duplicate runs.

To identify whether reduced expression of signalling proteins, in the presence of 4 pg/ml versus 40 pg/ml estradiol, was the result of diminished gene expression, the levels of mRNA for these signalling molecules were quantified by real-time PCR. RT-PCR data demonstrated that expressions of CD3-zeta, JAK2, and JAK3 mRNAs were significantly decreased in Jurkat cells incubated in 4 pg/ml estradiol compared to 40 pg/ml (Figure 6). Expression of CD3-zeta was 2.39-fold lower in cells exposed to 4 pg/ml compared to cells incubated with 40 pg/ml (p < 0.001). There was also diminished levels of gene expression for Janus kinases, JAK2 (2.01-fold) and JAK3 (2.21-fold), in T cells incubated in 4 pg/ml compared to those incubated in 40 pg/ml (p < 0.001). Gene expressions for CD3-zeta and JAK 3, incubated in 4 pg/ml E_2 _were not significantly different than that observed in cells exposed to 0 pg/ml (p > 0.05); while JAK 2 expression was significantly lower in 0 pg/ml E_2 _compared to 4 pg/ml (p < 0.05).

### Functional effect of estrogen on induction of IL-2 production

**Figure 7:**
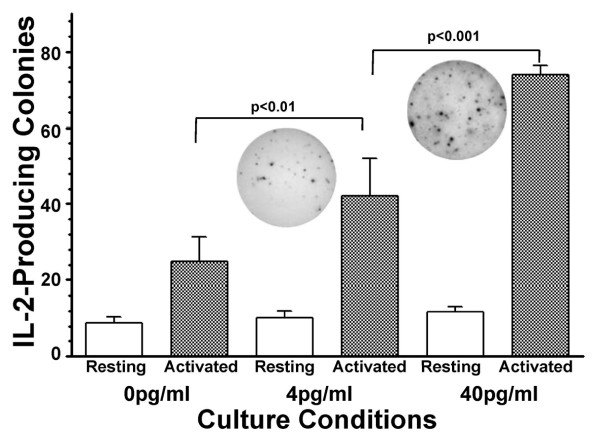
**Interleukin-2 (IL-2) production following induction by PMA and ionomycin in physiologic estradiol concentrations**. IL-2 production by Jurkat cells exposed to 4 pg/ml estradiol for 48 hours and activated with PMA and ionomycin for 24 hours compared to cells exposed to 40 pg/ml estradiol and activated. The number of IL-2 producing colonies was determined by ELISPOT assay and the values plotted are mean ± standard deviation. The inset images present representative assay plates for activated Jurkat cells in the presence of either 4.0 pg/ml E_2 _or 40.0 pg/ml E_2_.

Since expressions of components associated with T cell signalling were significantly lower in T cells incubated in 4 pg/ml estradiol, versus 40 pg/ml, are reduced levels of activation signalling components sufficient to alter the function of T cells? We defined the functional capacity of T cells as the induction of IL-2, which has been demonstrated to be diminished in postmenopausal women. Jurkat cells exposed to 4 pg/ml estradiol and activated with PMA and ionomycin exhibited significantly fewer IL-2-producing colonies of cells, when compared to Jurkat cells exposed to 40 pg/ml estradiol and activated, 50.7 ± 4.9 vs. 73.7 ± 2.6 colonies, respectively (p < 0.0001, Figure 7). Basal levels of IL-2 production were not significantly different in Jurkat cells, regardless of exposure to 0, 4 or 40 pg/ml estradiol (p = 0.0937), but the numbers of IL-2 colonies, following activation, with cells exposed to 4 pg/ml were significantly greater than that observed with 0 pg/ml estradiol (50.7 ± 4.9 versus 32.2 ± 6.9, p < 0.01).

## Discussion

A decline in the production of estrogens after menopause affects various immune parameters. Prior to menopause, both the cellular and humoral immune responses of women are superior to those of men, at all ages [24]. However, compared to premenopausal women, both men and postmenopausal women exhibit diminished lymphocyte proliferation and increased incidence of antibodies against self-antigens [7,9,30,31]. Previous studies have reported that menopause is associated with systemic and local changes in T and B cell subpopulations and function [24], as well as immunoregulatory cytokine production [32]. Hormone therapy has been used to prevent or decrease menopause-related symptoms and has been reported to affect T cell reactivity [26].

A chief immunological hallmark of aging is reduced T cell proliferation [26]. In women, this blunted immune response is likely to be the consequence of deficient estrogen production [1-7,25-27]. This reduced T cell proliferation has been shown to be a predictor of early mortality in the elderly [33]. Since induction of T cell proliferation is induced by cross-linking the TCR/CD3 complex and transducing the signal intracellularly through CD3-zeta, the lower expression of CD3-zeta in the presence of 4 pg/ml estradiol (compared to 40 pg/ml) observed in Figure 1 is consistent with the observation of diminished T cell proliferation in postmenopausal women. There are two major phosphorylated intermediates of CD3-zeta that form upon T cell activation, which migrate at 21 kDa and 23 kDa. It has been shown that the 21-kDa form is constitutively phosphorylated on the two membrane-distal ITAMs in thymocytes and peripheral T cells and it associates with nonphosphorylated ZAP70 as a result of *in situ *TCR interactions with peptide/MHC complexes. The 21 kDa intermediate can become phosphorylated at the most membrane-proximal ITAMs giving rise to the 23 kDa form. In this study, we demonstrate that, not only is the level of CD3-zeta decreased in cells exposed to 4 pg/ml E2, but the phosphorylation of the CD3-zeta protein chains is significantly diminished (Figure 3). Since this zeta phosphorylation is critical for the transduction and amplification of the activation signal, these consequences of low E2 would lead to a blunted response.

Similarly, Janus kinases (JAK-1,-2,-3 and Tyk-2) play important roles in signal transduction from cytokine receptors to the nucleus after phosphorylation of JAKs takes place following the binding of cytokines. Thus, reduced expression of JAK 2 (Figure 4) and JAK 3 (Figure 5) in the presence of 4 pg/ml estradiol is consistent with reduced signal transduction through cytokine receptors observed in postmenopausal women. The lower levels of CD3-zeta, JAK 2, and JAK3, resulting from exposure to 4 pg/ml estradiol, appears to be the result of decreased gene expression (Figure 6). The levels and expressions of these signalling components appear to be enhanced by the presence of estradiol, since their levels in both 4 pg/ml and 40 pg/ml estradiol were significantly elevated above those observed at 0 pg/ml.

In this *in vitro *model, we demonstrated that T cells exposed to 4 pg/ml estradiol (corresponding to a mean postmenopausal level) produced significantly less IL-2 than the identical cells at 40 pg/ml estradiol (premenopausal levels) (Figure 7). This is consistent with the observation of decreased production of anti-inflammatory cytokines, including IL-2, by lymphocytes from postmenopausal women. Our results not only confirmed that T-cells in low estrogen produced less IL-2 than T-cells in an estrogen environment analogous to premenopausal levels, but also established that this blunted immune response resulted from lower expression of signalling molecules, CD3-zeta, JAK 2, and JAK 3 in 4 pg/ml estradiol, versus 40 pg/ml. These signalling molecules are integral to the propagation of signals in T cells from activation by antigens and IL-2 [8,31,32]. Higher expressions of these signalling molecules in cells incubated with 40 pg/ml would be expected to exhibit an augmented response to receptor activation resulting in increased proliferation and production of Th1 cytokines in premenopausal women. The corollary of this would be that the decreased expression of signalling molecules associated with exposure to 4 pg/ml might account for the diminished proliferative capacity of T cell in postmenopausal women and the subsequent reduction in production of IL-2.

When the TcR/CD3 complex is exposed to premenopausal E_2 _levels (40 pg/ml) and activated with PMA and ionomycin, IL-2 is produced through a cascade of signalling events. IL-2 is released from the T cell and can act in a paracrine and autocrine effect to proliferate an immune response. IL-2 activates the IL-2 receptor and causes Janus kinases to autophosphorylate and lead to T cell proliferation and activation. However, when the TcR/CD3 complex is exposed to postmenopausal E_2 _levels (4 pg/ml) and activated with PMA and ionomycin, there is decreased production of CD3-zeta and Janus kinases, which leads not only to diminished IL-2 production, but also to poor signal transmission. Decreased IL-2 production is accentuated by poor transmission of IL-2 receptor signals as a result of decreased expressions of Janus kinases expressed in lower estrogen.

## Conclusion

While immunosenescence affects both men and women, the T cell functionalities of men, regardless of age, are less than that observed in premenopausal women and in general, do not exhibit the same age-related declines observed in women [34]. It has been suggested that, at least in terms of bone preservation, vascular function, and the central nervous system, local tissue levels of estrogens rather than circulating levels are the critical determinants [35,36]. A major physiological role for androgens in men (and postmenopausal women) may be as a circulating pool for local tissue estrogen production. In adipose tissue, vascular endothelium, bone and brain, aromatase converts androgens to estrogens. In contrast to the ovaries, extragonadal tissues lack the capacity to synthesize C19 steroids and thus their generation of adequate local estrogens depends upon the availability of C19 precursors [35,36]. Since the decline in circulating testosterone with age in men is relatively small and their levels are an order of magnitude greater than that observed in postmenopausal women (12 nmol/L in men vs. 0.6 nmol/L in postmenopausal women), several studies have indicated that circulating testosterone in men can be efficiently converted to estrogens in the extragonadal tissues to produce local estrogen concentrations sufficient to activate estrogen receptors [35,36]. In contrast, for women, there is a significant age-dependent decline in androgens, in addition to the decline in ovary-derived estrogens and together these may lead to traditional pathologies of estrogen deficiency [37]. Further, at the molecular level, studies have shown that estrogen receptor activation is distinct between men and women, as women exhibit a significantly distinct level of ER-associated coactivators [38]. It should be noted that, in studies using estrogen-containing therapies (unopposed estrogen or estrogen plus progestins), reversal of T cell unresponsiveness was observed [26,27,39]. These findings point to the critical role of circulating estrogens (or estrogen precursors) in maintaining immune responses in women.

Diminished levels of estrogens associated with menopause could lead to a blunted immune response that could increase susceptibility to pathogens and lead to higher incidences of mortality [33,40]. Previous studies reported that postmenopausal women display a greater prevalence of sepsis [41,42]. Clearly, our studies will need to be expanded to longer incubation periods, multiple estradiol concentrations and inclusion of additional estrogens, such estrone. Our current studies are addressing these observations *in vivo *using pre- and post-menopausal women, as well as women undergoing hormone therapy; however, the role of a single determinant cannot be appropriately evaluated using this approach and the number of signalling components and functionality will also be limited. Under physiologic conditions, transcriptional activation of the IL-2 gene by estrogens can occur and by binding to cell surface and intracellular receptors, estrogens can act synergistically with ligands to enhance IL-2 transcription [43]. Discovering the mechanisms by which the immune system may be blunted in an estrogen-deficient state may lead to future therapies, to aid postmenopausal women in becoming less prone to pathogens and to prevent the development of autoimmune and neoplastic diseases.

## Methods

### Cell lines

Jurkat E-6.1 cells, established from a human T cell lymphoma, were obtained from the American Type Culture Collection (Rockville, MD). This T cell line was routinely grown in RPMI 1640 medium supplemented with 10% fetal bovine serum, 0.1 mM nonessential amino acids, 1 mM sodium pyruvate, 200 mM L-glutamate, 100 μg/ml streptomycin and 100 IU/ml penicillin in a humidified 5% CO_2 _chamber at 37°C. Prior to estrogen studies, cells were grown in RPMI 1640 medium without phenol red but containing 10% charcoal-treated fetal bovine serum for a minimum of 72 hours. Subsequently, Jurkat cells were incubated in either 4 pg/ml or 40 pg/ml estradiol (E_2_) for 48 hours. Based on recent studies, the 4 pg/ml estradiol level was used to correspond to the postmenopausal levels (midpoint within the observed postmenopausal range, [44]), while 40 pg/ml estradiol represents a level associated with the mean of follicular and luteal phases of premenopausal women [45]. Cell viability was evaluated by trypan blue exclusion and all cultures utilized for this study were > 95% viable. All methods were performed in triplicate or quadruplicate to ensure reproducibility and accuracy.

### Patient samples

Heparinized blood specimens (10 ml) were obtained from premenopausal (cycling) women who were not pregnant and postmenopausal women, under an informed consent approved by the University Human Studies Committee of the University of Louisville. All blood samples were obtained from volunteers in the private office and clinics of the Department of Obstetrics and Gynecology at the University of Louisville. For premenopausal women, the mean age was 29.4 ± 3.8, while for postmenopausal women, the mean age was 54.4 ± 1.8. To assess, the level of CD3-zeta signalling molecules, T cells were isolated by the blood samples by magnetic activated cell sorting (MACS). Normal human PBMC were obtained from normal donors by isolation on Ficoll-Hypaque gradients. Untouched CD3-positive cells were obtained from the PBMC population by using a pan T cell isolation kit (Miltenyi Biotec, Auburn, CA) and the manufacturer's instructions.

### Expression of signalling proteins, CD3-zeta, JAK2 and JAK3

The expressions of CD3-zeta, JAK2, and JAK3 proteins were quantified by Western immunoblotting. Jurkat cells or normal T cells incubated with media containing either 4 pg/ml or 40 pg/ml of E_2_. After 48 hours, the cells were centrifuged and the cell pellet was washed and used for protein analysis. To assess for the expressions of T cell signalling proteins, the cell pellet was lysed using 50 mM HEPES (pH 7.2), 150 mM NaCl, 5 mM EDTA, 1 mM sodium orthovanadate, 2.5% Triton X-100, 200 μg/ml trypsin/chymotrypsin inhibitor, 200 μg/ml chymostatin, and 2 mM PMSF. The cell lysate was assayed for protein by the Bio-Rad protein assay. The modulation of signalling proteins was analyzed by Western immunoblot using a 15% SDS-PAGE gel. The proteins were electrophoretically separated by the method of Laemmli [46], analyzed by Western immunoblot as previously described [47], and probed overnight at 4°C with mouse monoclonal anti-CD3-zeta, anti-JAK2 and anti-JAK 3 antibodies (Santa Cruz Biotechnology, Santa Cruz, CA) as the primary antibodies. As an additional loading control, blots were probed using rabbit polyclonal anti-GADPH (Santa Cruz Biotechnology, Santa Cruz, CA). The presence of reactive bands was identified by incubation with peroxidase-conjugated anti-mouse immunoglobulins as the secondary antibody. The bound immune complexes were visualized by enhanced chemilluminescence (ECL, Amersham Biosciences, GE Healthcare, Piscataway, NJ) and quantitated by densitometry (Un-Scan-it software; Silk Scientific, Orem, UT).

### CD3-zeta activation

To define a functional consequence of estrogen levels corresponding to postmenopausal levels, initially the transduction of an activation signal was assayed as phosphorylation of CD3-zeta. Activation by TcR-CD3 ligation was performed by binding of 1 μg/ml anti-CD3 monoclonal antibody, UCHT-1 (functional grade, eBioscience, San Diego, CA) to Jurkat cells at 5 × 10^5^/ml for 15 min at 4°C. Additional cross-linking was achieved by incubating these cells with rabbit anti-mouse Ig (4 μg/ml) for 5 minutes at 37°C. Cells were then lysed in Tris-buffered saline, pH 7.2, 0.2 mM EDTA, 0.5% Triton X-100, 0.5 mM DTT, 20 mM β-glycerophosphate, 1 mM sodium orthovanadate, 5 mM sodium fluoride, 10 mM PMSF, 1 μg/ml leupeptin, and 1 μg/ml aprotinin for 30 min at 4°C. Lysates were centrifuged at 14,000 × *g *for 10 min at 4°C and supernatants were pre-clarified with protein A-Sepharose for 1 hour at 4°C. To isolate CD3-zeta protein, the clarified lysates (60 μg protein for Jurkat cells exposed to 40 pg/ml and 90 μg for those incubated with 4 pg/ml to correct for the diminished levels of CD3-zeta protein) were incubated with agarose-conjugated anti-CD3-zeta (Santa Cruz Biotechnology) with constant mixing overnight at 4°C. The complexes were pelleted by centrifugation at 10,000 × g for 10 minutes and the pellets washed with Tris-buffered saline containing 1% Triton X-100. The immunoprecipitates were analyzed by SDS-PAGE and immunoblotting using the mouse anti-human p-CD3-zeta (C415.9A) antibody (Santa Cruz Biotechnology, Santa Cruz, CA). The bands were analyzed as described above.

### Induction of IL2 production

To assess the consequence of estrogen on T cell function, induction of IL-2 was used as a marker. Production of IL-2 was analyzed using an enzyme-linked immunosorbent spot assay (ELISPOT) (Pierce Chemical Co., Rockford, IL). Ninety-six-well ELISPOT plates pre-coated with antibodies for IL-2 were used. Jurkat cells (10^6 ^cells/ml) were added to each well and incubated with and without 50 ng/ml PMA and 1 μg/ml ionomycin for 24 hours. After 24 hours, plates were emptied and washed twice with PBS and twice with PBS containing 0.05% Tween 20. Biotinylated anti-human IL-2 monoclonal antibodies, diluted to 1 μg/ml, were added and plates were incubated for 2 hours at room temperature. The contents were then discarded, the wells washed, and diluted Streptavidin-alkaline phosphatase solution was added to each well. The color reaction developed at room temperature in the dark for approximately 20 minutes. The plates were rinsed with distilled, deionized water, and allowed to dry upside down at room temperature for 60–90 minutes prior to analysis. Once the membrane was dry, the number of IL-2 secreting colonies, indicated as red spots, was manually enumerated using a dissection microscope and the median value of the quadruplicate wells calculated.

### CD3-zeta, JAK 2, and JAK 3 gene expression quantification

Real time-polymerase chain reaction (real-time PCR) was performed to quantify CD3-zeta, JAK2, and JAK3 gene expression. RNA was isolated using the TRIZOL reagent. The concentration of RNA was determined by measuring the absorbance in the spectrophotometer at 260 nm (*A*_260_) and 280 nm (*A*_280_). The purified RNA was stored at -70°C. cDNA was prepared from isolated RNA samples. This procedure was performed by the ReactionReady First Strand cDNA synthesis kit, as described by the manufacturer's instructions (SuperArray Bioscience Corp, Frederick, MD). cDNA was added to the real-time PCR mixture containing SYBR green and appropriate primer set. Specific primers for CD3-zeta, JAK2, and JAK3 were obtained from SuperArray Bioscience Corporation (Frederick, MD). Primers for GAPDH were included as an internal control. All determinations were performed twice with the LightCycler 2 (Roche Diagnostics, Indianapolis, IN). Data analysis was performed with the standard curve method. Since mRNA level is defined following its transcription into cDNA, the levels of gene expression are presented as cDNA concentration.

### Statistical Analysis

Incubations of Jurkat cells with various E_2 _concentrations were performed in three several experiments. Western blot analysis of each signaling protein was performed in duplicate from each individual experiment. Western blot images for CD3-zeta, JAK2, and JAK3 were digitized and statistical analysis performed on the digitized images using the one-way ANOVA with Bonferroni's multiple comparison test. RNA was isolated from each condition from each of the three separate studies. RT-PCR quantitations of mRNA levels were performed in duplicate. ELISpot analysis was performed on cells from each of the 3 separate experiments and each E_2 _concentration was determined in quadruplicate for each ELISpot assay. Statistical analysis of Il-2 production and quantification of cDNA through real-time PCR was performed using the student's t-test. A p value of < 0.05 was considered statistically significant.

## Competing interests

The authors declare that they have no competing interests.

## Authors' contributions

Each of the authors has made substantive intellectual contributions to the conception and design of this study, as well as in the acquisition, analysis and interpretation of the data. Each author was involved in the drafting of the manuscript and given final approval to this version.
